# Extracellular vesicles from bacteria and fungi: mechanistic insights and implications for urinary tract infections

**DOI:** 10.20517/evcna.2025.49

**Published:** 2025-11-28

**Authors:** Eddie Chung-Ting Chau, Pak-Ting Hau, Michaela Murillo, Chi-Ching Tsang, Emily Wan-Ting Tam, Sai-Wang Seto, Cheuk-Lun Lee, Franklin Wang-Ngai Chow

**Affiliations:** ^1^Department of Health Technology and Informatics, The Hong Kong Polytechnic University, Hong Kong, China.; ^2^School of Medical and Health Sciences, Tung Wah College, Hong Kong, China.; ^3^School of Science and Technology, Hong Kong Metropolitan University, Hong Kong, China.; ^4^School of Biomedical Sciences, The University of Western Australia, Perth 6009, Australia.

**Keywords:** Urinary tract infections, extracellular vesicles, host-pathogen interaction, antimicrobial resistance

## Abstract

Urinary tract infections (UTIs) pose a significant public health challenge, affecting approximately 407 million people worldwide and causing substantial morbidity and approximately 237,000 deaths. Bacteria and fungi represent the most frequent causative microbes, leading to symptoms such as low abdominal pain, fever, frequent urination, hematuria, sepsis, inflammation of the bladder and kidney, and even death. In recent years, extracellular vesicles (EVs) have emerged as critical mediators of UTI pathogenesis. EVs are lipid bilayer nanoscale particles that carry DNA, RNA, enzymes, and other biomolecules. They can facilitate microbial colonization, immune modulation and evasion, tissue invasion, and antimicrobial agent resistance. This review summarizes current knowledge on the role of bacterial and fungal-derived EVs in UTIs, their mechanisms of action, and their potential therapeutic implications.

## INTRODUCTION

Urinary tract infections (UTIs) remain a significant public health challenge worldwide. In 2019, approximately 407 million people were affected, resulting in approximately 237,000 deaths^[1]^. UTIs can be caused by highly diverse microbes, including fungi, bacteria, parasites, and viruses, and the symptoms can range from dysuria and low abdominal pain to fever, frequent urination, and hematuria^[2]^. Severe complications include sepsis, renal inflammation, and organ failure, as well as multi-organ dysfunction^[3]^. Of note, recurrent infection is widespread^[4]^.

**Figure 1:**
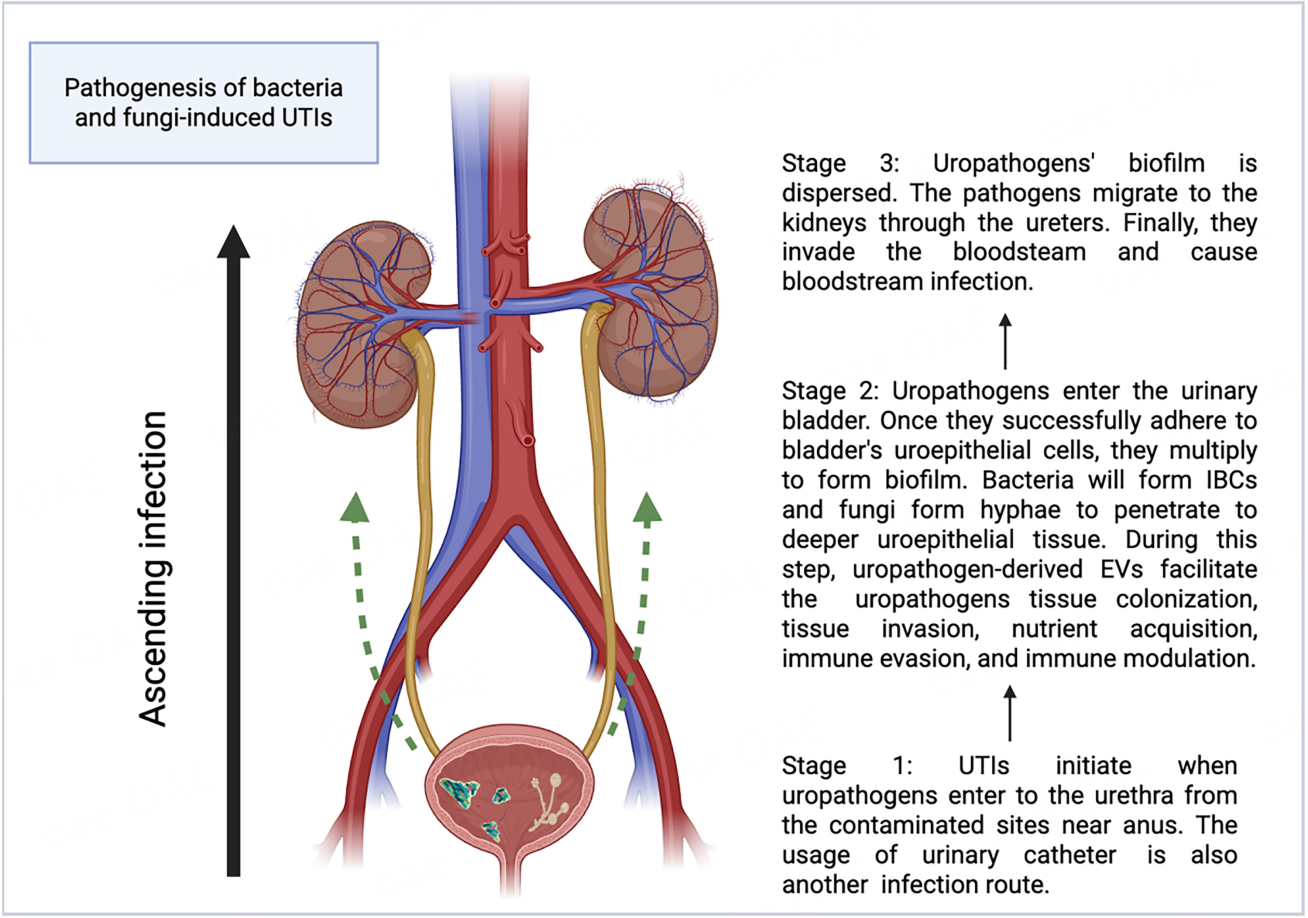
Pathogenesis of bacteria- and fungi-induced UTIs (Created in Biorender). UTIs can be divided into three stages. In the first stage, uropathogens invade and colonize the urethra^[5-7]^. In the second stage, they invade the urinary bladder and form biofilms^[11,12,20-22]^, where uropathogen-derived extracellular vesicles (EVs) facilitate invasion, colonization, nutrient acquisition, and immune modulation and evasion^[13-19,27-33]^. The third stage involves biofilm dispersion and propagation of uropathogens to the kidneys via the ureters and eventually to the bloodstream, causing systemic infection^[12,24-26]^. Created in BioRender. Chau, C. (2025) ( https://BioRender.com/st81pck). UTIs: Urinary tract infections; EVs: extracellular vesicles; IBCs: intracellular bacterial communities.

UTIs typically start when pathogens enter from the anus or a contaminated area and ascend the urethra to the urinary bladder^[5]^. Successful urethral colonization is a prerequisite for UTIs^[6]^. Pathogens utilize their flagella to migrate from the urethra to the urinary bladder^[7]^. Adhesins are secreted to counteract the urine flow and facilitate attachment to receptors on uroepithelial cells^[8,9]^. Urinary catheters provide an additional route for pathogen entry into the bladder^[10]^. Once inside the bladder, pathogens attach to the uroepithelial cells and begin to replicate^[11,12]^. In the meantime, uropathogens adopt various strategies to evade and modulate the host immune response^[13-19]^. Bacteria, such as uropathogenic *Escherichia coli* (UPEC), hide in and enter the uroepithelial cell cytosol to form intracellular bacterial communities (IBCs)^[20]^. Fungi, especially *Candida albicans* (*C. albicans*), form hyphae and secrete agglutinin-like sequence 3 protein (Als3) for the uroepithelial cell attachment, which is critical for biofilm formation and active penetration into the host’s deeper uroepithelial cells and further into the bloodstream^[21,22]^. Furthermore, uropathogens produce toxins and express proteins for survival in the bladder^[14,23]^. Upon successfully colonizing the urinary bladder, uropathogens can be dispersed from the biofilm and invade the kidneys through the ureters, ultimately causing bloodstream infection^[12,24-26]^. Figure 1 shows the pathogenesis of bacteria- and fungi-induced UTIs.

Treatment of UTIs currently relies primarily on antibiotics (e.g., trimethoprim sulfamethoxazole, ampicillin, and ciprofloxacin), antifungal drugs (e.g., fluconazole and flucytosine for *Candida* UTI), anti-parasitic drugs (e.g., praziquantel for treating urinary schistosomiasis), and anti-viral drugs (e.g., cidofovir for treating cystitis)^[34-36]^. However, the non-judicious use of these medications has led to the emergence of drug-resistant microorganisms, complicating the effective management of UTIs worldwide. This situation highlights the urgent need to study the drug-resistant mechanisms of uropathogens to identify new therapeutic targets, ultimately improving patient outcomes and addressing the challenge of drug-resistant UTIs^[37,38]^.

Despite clear identification of causative agents in many UTIs, the mechanisms by which different microbes interact with the host and survive antimicrobial exposure still need further investigation. Recently, microbial-derived extracellular vesicles (EVs) have gained increasing attention. These lipid bilayer particles act as messengers between microbes and hosts by carrying metabolites, proteins, DNA, and RNA^[39]^. Compared to human-derived EVs, microbial-derived EVs differ substantially in biogenesis, composition, and function (except for fungi), which has attracted interest in their role during infections.

Regarding EV biogenesis, humans and fungi both generate exosomes via inward budding through the ESCRT (Endosomal Sorting Complex Required for Transport) pathway and ectosomes via outward budding^[40-46]^. The ESCRT pathway involves several components, including ESCRT-0, I, II, and associated proteins such as Alix^[45-47]^. First, ESCRT-0’s HRS (hepatocyte growth factor-regulated tyrosine kinase substrate) binds phosphatidylinositol-3-phosphate (PI3P), recruiting ESCRT-0 to the endosomal membrane^[45-47]^. The HRS domain subsequently attracts ESCRT-I through the TSG101 domain^[45-47]^. ESCRT-II, together with ESCRT-I, deforms the membrane to cause inward budding^[45-47]^. ESCRT-III will then undergo vesicle scission and promote the formation of intraluminal vesicles (ILVs) in the multivesicular bodies (MVB), which will then be released as exosomes^[45-47]^. However, for the outward budding, the ectosome is formed via the outward budding action of the cell membrane^[45]^. Of note, the possibility of the presence of other EVs biogenesis pathways of fungi that are different from that of humans may exist.

In contrast, gram-negative bacteria produce EVs (outer membrane vesicles and outer inner membrane vesicles) through outer membrane blebbing and explosive cell lysis^[48,49]^. In outer membrane blebbing, the EVs are formed through the blebbing of the bacterial outer membrane^[48,50]^. For explosive cell lysis, the membrane vesicles are formed from the lysis membrane fragments, as a result of stress-induced bacterial cell lysis^[50,51]^. Nonetheless, the EV production pathway of gram-positive bacteria is still uncertain, which leaves a research gap for scientists to study the components inside the bacterial cell that contribute to EV production^[52]^.

Regarding the EVs’ composition and function, human-derived EVs deliver proteins, lipids, DNA, and RNA essential for maintaining homeostasis and involved in different pathological processes^[48,53,54]^. The main target of human-derived EVs is the human cell, for example, immune cells during the infection^[55]^. On the contrary, microbial-derived EVs carry proteins, lipids, DNA, and RNA that are important for facilitating their survival in the environment and invasion into the host^[56,57]^. It is of particular interest how these tiny vesicles can open a gateway to help microbes colonize and cause serious illness in human hosts, such as UTIs. Below, the differences between human and microbial-derived EVs (Fungi and bacteria) are shown in Table 1.

**Table 1:** Similarities and differences of EVs derived from humans, fungi, and bacteria

	**Humans**	**Fungi**	**Bacteria**	**References**
**Reported EV size (nm)**	30-400	50-400	50-500	[58-60]
**Production route**	Inward budding through ESCRT pathway and outward budding	Inward budding through ESCRT pathway and outward budding	Outer membrane blebbing and explosive cell lysis	[40-51]
**Common EV markers**	CD9, CD63, CD81, flottilin-1	Not yet defined	Not yet defined	[61]
**EV content**	DNA, RNA, protein, lipid	DNA, RNA, protein, lipid	DNA, RNA, protein, lipid, peptidoglycan	[48,56,57]
**Main function**	Homeostasis maintenance and immune defense	Survival in environment, facilitation of host infection and invasion	Survival in environment, facilitation of host infection and invasion	[54-57]

EV: Extracellular vesicle; ESCRT: endosomal sorting complex required for transport.

Besides, microbial-derived EVs are critical in enhancing microbes’ adherence to host tissue, biofilm formation, host immune response modulation, immune evasion, and decreasing antimicrobial agent susceptibility in the course of infection^[62-65]^. Few studies discussed how UTI pathogens interact with the host via EVs^[27,29,66]^. There is no doubt that EVs play a significant role during UTIs and minimize the effects of antimicrobial agents. In this review, we summarize the role of EVs derived from UTI-causing bacterial and fungal pathogens, thereby giving insight for microbiologists and clinicians to further study the UTI pathogen and host interaction. Additionally, Table 2 presents a brief summary to give readers an overview of the proteins or molecules in uropathogen-derived EVs that contribute to infection.

**Table 2:** Summary of the role of identified proteins/molecules in uropathogenic bacterial and fungal-derived EVs

**Characteristics of UTI’s pathogen/roles during UTIs**	**Involved protein in bacterial-derived EVs**	**Involved protein/molecules in fungal-derived EVs**	**References**
Colonization	AroB, AroG, AroK, MrpA	PHR1, XOG1, BGL2, CSH1, MP65, AMS1, Met6, TOS1, MNT1, CHT3, TRX2, SAP5, PET9	[27,29-32,76-79,105,107,108]
Invasion to the host’s bloodstream	Hemolysin, EfeO, FepA	Sap2, RAS1	[14,27,33,80,82,84-89,111,115-117]
Immune modulation/evasion	FimH, CNF1, LPS, Flagellin	Glucuronoxylomannan, Sap6	[13-19,27,95-98,122,127-129]
Antimicrobial agent resistance	A-band liposaccharide, KCP protein, Aac6’-le-Aph2”-la, Aph3’-III, VanR-A, VanS-A, VanH-A, VanA, VanX-A, VanY-A, VanZ-A, VanS-B, ErmB		[30,31,87,99,101]
Proposed protein that may contribute to antifungal agent resistance		xog-1-like, mp65-like and alcohol dehydrogenase 1	[65,130-134]

Aac6’-Ie-Aph2’’-Ia: Aminoglycoside N-acetyltransferase 6’-Ie/aminoglycoside O-phosphotransferase 2’’-Ia; Aph3’-III: aminoglycoside O-phosphotransferase type III; AroB: 3-dehydroquinate synthase; AroG: phospho-2-dehydro-3-deoxyheptonate aldolase; AroK: shikimate kinase; CHT3: chitinase 3; CNF1: cytotoxic necrotizing factor 1; CSH1: cell surface hydrophobicity protein 1; EfeO: iron transporter component EfeO; ErmB: erythromycin ribosomal methylase B; FepA: ferrienterobactin receptor; FimH: type 1 fimbrial adhesin; Flagellin: flagellar filament structural protein; LPS: lipopolysaccharide; Met6: homocysteine methyltransferase; MNT1: glycolipid 2-alpha-mannosyltransferase; MrpA: Mrp antiporter subunit A; MP65: 65 kilodalton mannoprotein; PET9: ADP/ATP carrier protein 2; PHR1: glycosylphosphatidylinositol-anchored beta(1,3)-glucanosyltransferase; RAS1: RAS-like protein 1; SAP2: secreted aspartyl proteinase 2; SAP5: candidapepsin-5; Sap6: secreted aspartyl proteinase 6; TOS1: probable circularly permuted 1,3-beta-glucanase; TRX2: thioredoxin 2; VanA: vancomycin resistance protein A; VanH-A: vancomycin resistance protein H-A; VanR-A: vancomycin resistance regulator A; VanS-A: vancomycin resistance sensor A; VanS-B: vancomycin resistance sensor B; VanX-A: vancomycin resistance D,D-dipeptidase; VanY-A: vancomycin resistance D,D-carboxypeptidase; VanZ-A: vancomycin resistance protein Z-A; XOG1: glucan 1,3-beta-glucosidase; BGL2: β-1,3-glucanosyltransferase; AMS1: α-mannosidase; A-band liposaccharide: major O-antigen polysaccharide; KCP protein: *K. pneumoniae* carbapenemase (KPC) protein; Glucuronoxylomannan: polysaccharide capsule component of *Cryptococcus*.

## THE ROLE OF BACTERIA-DERIVED EVs IN BACTERIAL UTIs

Gram-negative bacteria, such as *Escherichia coli* and *Klebsiella spp*, are UTIs’ most common causative agents^[67]^. Although the human urinary tract has several intrinsic antimicrobial mechanisms, such as the secretion of glycoproteins that block microbial adherence to the uroepithelium, bacteria have a “secret strategy”, EVs, to overcome these defense mechanisms^[68]^.

### Bacteria-derived EVs facilitate movement and biofilm formation

Biofilm formation and motility are critical factors for bacterial colonization in urinary tissue and urinary catheters during UTIs^[69,70]^. Studies have shown that EVs derived from UPEC clinical isolate PMH can enhance motility and biofilm formation, potentially affecting the motility-to-biofilm transition pathway^[29]^. A key regulator molecule of this pathway, bis-(3’-5’)-cyclic dimeric guanosine monophosphate (c-di-GMP), is upregulated during biofilm formation; then, it interacts with the protein YcgR (commonly known as flagellar brake)^[71,72]^. The interaction between these molecules further leads to the direct contact of YcgR to the flagellar motor proteins FliG and MotA, thereby inhibiting the motility of the bacteria for biofilm formation^[72]^. Additionally, to facilitate the dispersion of bacteria’s biofilm in the urinary tract, a carbon storage regulator, CsrA, is gradually released during biofilm formation, whose primary function is to disaggregate the biofilm and facilitate the propagation of bacteria in the urinary tract^[73,74]^. However, further investigation is needed to confirm the role of EVs in the motility-to-biofilm transition pathway and to identify which components are affected by the bacterial-derived EVs. Previous research has demonstrated a strong relationship between the c-di-GMP level and bacterial EV synthesis, which in turn affects biofilm formation^[75]^. It would be interesting to determine whether EVs function as downstream effectors or regulators within this pathway. Alternatively, aromatic amino acid (AAA) synthesis proteins [3-dehydroquinate synthases (AroB), Phospho-2-dehydro-3-deoxyheptonate aldolase, Phe-sensitive (AroG), Phospho-2-dehydro-3-deoxyheptonate aldolase, Trp-sensitive (AroH), and shikimate kinase I (AroK)] have been identified in the EVs of *E. coli*^[29]^. A recent study reported a significant reduction of motility in *E. coli* mutants lacking AroB, AroG, and AroK, while the addition of EVs derived from the wild type successfully restored the motility of the AroB mutant. This finding implies that *E. coli*-derived EVs can transfer to other bacterial cells and facilitate their migration along the urinary tract^[29,76]^. In addition, EVs from another uropathogen, *Proteus mirabilis*, contain MR/P fimbriae structural component (MrpA), which is involved in the attachment of uroepithelial tissue^[27,77-79]^. Altogether, these findings indicate that bacterial EVs play an extensive role in promoting bacterial motility and tissue colonization during UTIs.

### EVs facilitate persistent bacterial infection and invasion

Another well-studied human urinary tract defense mechanism is iron depletion. Iron serves as an essential enzyme cofactor in bacterial survival during colonization^[80,81]^. In humans, most iron is complexed with heme groups and found in the hemoglobin of erythrocytes^[82]^. Meanwhile, iron released into plasma is bound to transferrin, limiting bacterial access to free iron^[82,83]^. Despite this, bacteria can acquire iron from the host during infection. One strategy is to secrete hemolysin, which is believed to lyse host cells and release nutrients and minerals, such as iron, during UTIs^[82,84-86]^. A recent study has illustrated that the iron content in the urine of healthy individuals is lower than that of UTI patients^[85]^. This suggests a sign of iron extraction in the urinary system. In this process, EVs derived from uropathogens, such as *Proteus mirabilis*, UPEC, and *Pseudomonas aeruginosa*, are responsible for delivering hemolysin to the host cell and causing urinary tissue damage, including uroepithelium shedding and bladder hemorrhage^[27,33,87,88]^. Furthermore, the iron acquisition system components, such as ferrienterobactin receptors FepA and EfeO (an iron-binding protein), were found in the EVs derived from *E. coli*^[27,33,89]^. FepA is an outer membrane receptor that facilitates the transport of iron-siderophore complexes into bacterial cells^[80]^. Inside the bacterial cytoplasm, iron-enterobactin esterase releases iron from the siderophore complex by cleaving the enterobactin backbone^[80]^. It is possible that FepA in bacterial EVs can fuse the membranes of other bacterial cells and increase iron uptake. This concept is supported by a study that discovered the translocation of receptors from EVs to the cell membranes of other cells^[90]^. Additionally, EfeO is a membrane protein responsible for transporting ferric ions into bacterial cells^[91]^. It has also been found to play a role in maintaining iron homeostasis in the cells by facilitating the oxidation of excess ferrous ions to ferric ions^[92]^. Altogether, this highlights that EVs facilitate the invasion and survival of uropathogens in the nutrient-resource-limited urinary system after or during colonization.

### Bacteria-derived EVs promote immune modulation and evasion

Immune evasion is also significant in maintaining the persistent colonization and invasion of uropathogenic bacteria. Although few studies have described the relationship between bacteria-derived EVs and immune evasion during UTIs, some general mechanisms that explain the interaction between bacteria-derived EVs and the host immune system are relevant. For example, Tomasek *et al*. have discovered that the FimH protein, a component of type 1 pili in UPEC, can bind with CD14 expressed on dendritic cells and inhibit the dendritic cell migration to lymph nodes^[13]^. This is achieved by overactivation of the integrins and nuclear factor of activated T-cells pathway, thus suppressing the immune response^[13]^. In addition, Dadi *et al*. have reported that the FimH gene was highly expressed in the UPEC isolated from the patient’s urine^[93]^. Furthermore, EVs derived from the UPEC cultured in a Luria broth (LB) medium contain FimH, illustrating the role of adhesin delivery in bacterial EVs^[27]^. Nevertheless, the presence of FimH in the EVs of uropathogenic bacteria during UTIs requires further investigation and confirmation. Moreover, it is of great interest to know if the FimH protein in *E. coli*-derived EVs can bind directly to CD14 on dendritic cells and prevent its migration. Of note, cytotoxic necrotizing factor type 1 (CNF1) is another virulence factor that is proven to be delivered by the EVs of UPEC^[18]^. CNF1 is found to downregulate CD36 transcription by decreasing the CD36 transcription factors [liver X receptor β (LXRβ) and cytosine-cytosine-adenosine-adenosine-thymidine/enhancer binding protein α (C/EBPα)], thereby inhibiting macrophage phagocytosis and reducing uropathogen clearance in the urinary system^[19]^. Furthermore, UPEC-derived EVs were shown to induce mitochondrial dysfunction in macrophages, which inhibited the production of anti-apoptotic protein, myeloid cell leukemia-1 (MCL-1), and triggered cell death of macrophages, leading to the further spread of the pathogens in the urinary system^[94]^. Overall, bacterial EVs increase their chances of survival by promoting immune evasion during UTIs. In addition to immune evasion, bacteria-derived EVs carry pathogen-associated molecular patterns (PAMPs), which have been demonstrated to initiate inflammatory immune responses in some non-UTIs studies^[95,96]^. They are present both on the surface of and within EVs, including lipopolysaccharide (LPS) and flagellin^[95-98]^. Flagellin was shown to induce caspase recruitment domain (CARD) domain-containing 4 (NLRC4) canonical inflammation and upregulate interleukin (IL)-1β production^[97]^. Moreover, a study has shown that EVs are essential components for delivering LPS to the cytosol of bone-marrow-derived macrophages (BMDM), which in turn triggers caspase-11 activation and inflammasome production^[98]^. This shows that EVs themselves can activate immune responses, apart from promoting immune evasion.

### Bacteria-derived EVs promote antibiotic resistance

Bacteria-derived EVs play a critical role in inducing antibiotic resistance. For instance, EVs from a uropathogen, *Pseudomonas aeruginosa*, increased the minimum inhibitory concentration (MIC) of gentamicin 4-fold, and A-band LPS was present only in gentamicin-induced EVs but not in natural EVs^[87]^. This may be the critical component contributing to drug resistance. Another uropathogen, carbapenem-resistant *Klebsiella pneumoniae*, has been shown to secrete EVs to hydrolyze meropenem, and the authors speculate that the *K. pneumoniae* carbapenemase (KPC) protein in EVs contributes to the hydrolysis effect in meropenem^[99]^. Furthermore, carbapenem-resistant hypervirulent *K. pneumoniae* was shown to secrete EVs to deliver the drug-resistant and virulence plasmid to the less virulent *K. pneumoniae*, showcasing that EVs can facilitate horizontal gene transfer between the same species and protect bacterial plasmids from degradation^[100]^. In addition, EVs derived from *Enterococcus faceium* were shown to consist of proteins that are associated with resistance to aminoglycoside (Aac6’-le-Aph2”-la, Aph3’-III), glycopeptide (VanR-A, VanS-A, VanH-A, VanA, VanX-A, VanY-A, VanZ-A, VanS-B), and macrolide (ErmB), suggesting the significant role of uropathogenic bacteria-derived EVs in promoting antibiotic resistance^[101]^.

## ROLE OF FUNGAL-DERIVED EVs IN FUNGAL UTIs

Fungi utilize EVs to strive for persistent survival and propagation in the urinary system. A notable species is *C. albicans*^[102]^. There are two major infection pathways: entry of uropathogenic fungi from the bloodstream into the urinary system, or ascension from the urethra or a site near the urethra into the upper urinary tract^[103]^. Adhesion is the first stage in these pathways.

### EVs facilitate adhesion and colonization in the urinary system via biofilm formation and dispersion

Fungi-induced UTIs, particularly *C. albicans,* can commonly be found in chronically catheterized patients in hospitals due to the ability of the fungus to form hyphae and biofilm in the urinary tract and urinary catheter^[9,104]^. EVs derived from fungi play a vital role in this process. Recently, a study showed that the composition of EVs derived from *C. albicans* is highly similar to the matrix material for biofilm formation^[30,105]^. Later, three enzymes, Glucan 1,3-beta-glucosidase (XOG1), cell wall 1,3-beta-glucosyltransferase (BGL2), and glycosylphosphatidylinsitol-anchored beta(1,3)-glucanosyltransferase (PHR1), in the EVs from *C. albicans* were identified to be essential for delivering the beta-1,3 glucan (key components of biofilm) to the biofilm matrix and contributing to the mature biofilm mass^[30,31]^. Notably, EVs derived from *C. albicans* contain several proteins [e.g., PHR1, cell surface hydrophobicity protein 1 (CSH1), 65-kilodalton mannoprotein (MP65), XOG1, α-mannosidase (AMS1), homocysteine methyltransferase (MET6), circularly permuted 1,3-beta-glucanase (TOS1), glycolipid 2-alpha-mannosyltransferase (MNT1), chitinase 3 (CHT3), thioredoxin 2 (TRX2), candidapepsin-5 (SAP5), ADP/ATP carrier protein 2 (PET9)] involved in the endosomal sorting complexes required for transport (ESCRT) pathway. These cargo proteins, which can either promote the biofilm adhesion or biofilm dispersion, are selectively packaged into EVs of *C. albicans*^[32]^. This facilitates the adaptation of the uropathogenic fungus in the dynamic urinary system and the propagation of the fungus in the host, since it can adjust and control its behavior based on different environmental condition changes, for example, nutrient availability, the presence of immune cells and flow conditions, in the urinary system^[106]^. Apart from *C. albicans*, EVs of the other uropathogenic fungi, *Candida parapsilosis* (*C. parapsilosis*), and *Candida tropicalis* (*C. tropicalis*) comprise the cell wall mannoprotein MP65^[107,108]^. This protein is responsible for adhesion on the plastic surface and is essential for germ tube development during hyphae formation^[107,108]^. This phenomenon increases the risk of catheter-associated UTIs, particularly because some urinary catheters are made of plastic^[109]^.

### EVs derived from uropathogenic fungi promote invasion into the bloodstream in the urinary system

In addition to the characteristics of biofilm dispersion discussed previously, EVs derived from the fungus promote the yeast-to-hyphae transition in a study^[28]^. The transition process (or the dimorphism) mainly depends on the presence of nutrients and chemicals during the infection, such as glucose and trichloroacetic acid in the serum, the quorum-sensing molecules (e.g., farnesol), and the activation of the gene RAS-like protein 1 (RAS1)^[110-112]^. Research has shown that *C. albicans* uptake with EVs derived from the *C. albicans* hyphae state can promote more hyphae and pseudohyphae formation when compared with the EVs derived from the yeast form of *C. albicans* during their growth, and there is a depression of the CHT2 gene (endochitinase)^[28]^. The CHT2 gene was abundant in the yeast form of the fungus but not in its hyphae state^[113]^. Notably, hyphae are a significant virulence factor of fungi during UTIs, as they can penetrate the urinary endothelial cells and further into the bloodstream^[102]^. However, it remains unclear whether the EVs of *C. albicans* can promote both pseudohyphae and hyphae formation. Another study shows a contradictory result, in which they found that EVs from the yeast state of *C. albicans* favor the formation of pseudohyphae only, not hyphae^[114]^. The conflicting results may stem from different growing conditions or the state of the fungus when the EVs are obtained^[28]^. This assumption can be further supported by a study of Martínez-López *et al*. in which they discovered the presence of RAS1 protein inside the EVs extracted from the hyphae state of *C. albicans*, but not in the EVs of the yeast state^[14]^. RAS1 is a signal protein that drives the yeast to hyphae transition in *C. albicans*^[111,115]^. Deleting the RAS1 gene or inhibiting the RAS1 protein in *C. albicans* showed the failure of germ tube and hyphae formation, but this was not the case in pseudohyphae^[111,115]^. It is possible that EVs derived from the hyphae state of *C. albicans* deliver active RAS1 protein to the yeast state of *C. albicans*, which promotes the germ tube and true hyphae formation in the presence of D-glucose^[116]^. It has been discovered that D-glucose can act as a single stimulus that activates the RAS1, and the active RAS1 protein can further activate adenylyl cyclase (Cdc35), followed by enhanced filamentous growth 1 (Egf1) protein, to promote hyphae formation^[116]^. Altogether, this highlights that EVs derived from the hyphae state of *C. albicans* can promote invasion through the formation of true hyphae. Moreover, another strategy in the EVs derived from the fungi that can disrupt the endothelial layer is the enzyme aspartic protease 2 (Sap 2)^[117]^. This enzyme is responsible for the rupture of the vascular endothelial cell and lets the uropathogenic fungus gain access to the bloodstream, which ultimately causes disseminated candidiasis^[118,119]^.

### EVs derived from uropathogenic fungi cause immune modulation and evasion

The interaction between the immune systems of the hosts and fungi during UTIs has not been investigated extensively. Limited and non-UTI-related studies have suggested that EVs can act as double-edged swords in this case^[57]^. A recent non-UTIs study illustrated that EVs derived from the hyphae state of *C. albicans* caused cytotoxic effects on THP-1 macrophages^[40]^. However, other studies reported that EVs from *C. albicans* enhanced the killing effect in bone marrow-derived macrophages (BMDMs) but not in *Candida auris* (*C. auris*) MMC2 isolate, in which its EVs increased the survival rate within the macrophages^[120]^. The reason behind this is that EVs from *C. albicans* that are taken by the BMDMs upregulate the level of nitric oxide, IL-12p40, tumor necrosis factor-alpha, as well as the co-stimulatory molecules of macrophages and dendritic cells^[120,121]^. It is of particular interest to study further the effects of EVs from *C. albicans* in different types of macrophages, such as human urinary system macrophages. Moreover, another significant component in *C. albicans*’ EVs is aspartyl protease 6 (Sap6)^[14]^. It was shown that Sap6 could reduce the reactive oxygen species (ROS) production efficiency via the nicotinamide adenine dinucleotide phosphate (NADPH) oxidase degradation, once it was internalized in the neutrophils^[122]^. The degradation of NADPH oxidase inhibited the production of the neutrophil extracellular traps^[122]^. This further activated the proapoptotic caspases 3/7, thus promoting the neutrophil apoptosis^[122]^. Additionally, a recent study has found that DNA inside the EVs of *C. albicans* and *C. auris* triggered type 1 interferon signaling through cyclic guanosine monophosphate (GMP)-adenosine monophosphate (AMP) synthase (cGAS) - stimulator of interferon (IFN) genes (STING) pathway, thus increasing the fungal clearance activity in macrophages^[123,124]^. In brief, EVs from *C. albicans* and *C. auris* activate the cytosolic sensor, cGAS^[123]^. The activation of cGAS further leads to the synthesis of cyclic guanosine monophosphate-adenosine monophosphate (cGAMP)^[123,125,126]^. Then, the STING protein binds to cGAMP and undergoes polymerization^[125,126]^. The polymerized STING protein is translocated to the nucleus and activates Tumor Necrosis Factor Receptor-associated Factor family member-associated nuclear factor kappa B activator (TANK)-binding kinase 1 and interferon regulatory factor 3 (IRF3)^[125,126]^. Activated IRF3 triggers the gene expression of type 1 interferon to fight against the pathogen or pathogen-derived molecules^[125,126]^. Apart from *C. albicans*, EVs from a uropathogenic fungus, *Cryptococcus neoformans*, contain the polysaccharide glucuronoxylomannan, which exhibits the immunosuppressive effect via inhibition of T-cell response and impairs the fungicidal activity of neutrophils^[16,17,127-129]^. Furthermore, it has been demonstrated to induce apoptosis in macrophage RAW264.7 cells by upregulating the level of signal transducers and activators of transcription 1 (STAT1) and inducible nitric oxide synthase (iNOS)^[15]^. The elevation of iNOS subsequently led to apoptosis in RAW264.7 macrophages^[15]^. Overall, these display that EVs derived from different species or strains of fungi may have various immune modulation effects during UTIs.

### EVs from uropathogenic fungi promote antifungal drug resistance

Antifungal drug resistance is an urgent public health problem, and EVs derived from fungi are believed to contribute to this issue^[32,65]^. A study shows that back-addition of nano to microgram of EVs secreted from a uropathogenic fungus, *C. auris*, increased the MIC to amphotericin B (AMB) 16-fold, which the authors propose that the presence of high quantities of alcohol dehydrogenase 1 (Adh1), XOG1, and MP65-like (mannoprotein-65) protein in EVs may contribute to this resistant effect^[65]^. Adh1, an enzyme that manipulates alcohol production, is associated with biofilm formation and fluconazole resistance^[130]^. The efflux pump is the major mechanism of fluconazole resistance^[131]^. Researchers propose that Adh1 overexpression activates the glycolytic pathway, which increases adenosine triphosphate (ATP) production to support the efflux pump activity^[131]^. However, the relationship between AMB resistance and Adh1 is waiting to be established. Apart from that, the xog-1 protein is heavily involved in forming biofilm^[30,31]^. It has been previously shown that its expression in *C. albicans* was upregulated with the AMB treatment^[132]^. Moreover, MP65 is a beta-glucanase and its deletion mutant lost the ability to form biofilm^[133,134]^. Thus, it is important to determine whether this protein contributes to AMB and other antifungal drug resistance. Furthermore, another study has demonstrated that adding EVs to the *C. tropicalis* culture increased the thickness of the biofilm and its metabolic activity under the treatment of fluconazole and caspofungin^[135]^. Still, the fungal viability was only increased in the caspofungin group^[135]^. Additionally, an antifungal drug, turbinmicin, which inhibits the vesicle trafficking pathway in fungi, impaired *C. albicans*’ EVs delivery to the biofilm matrix and increased the fluconazole susceptibility in the [(sodium 3′-[1- (phenylaminocarbonyl)- 3,4- tetrazolium]-bis (4-methoxy6-nitro) benzene sulfonic acid hydrate)] (XTT) assay^[136,137]^. The vesicle trafficking pathway inhibition by turbinmicin is due to the binding of a protein, Sec14 (yeast phosphatidylinositol transfer protein), and the hindrance of the accumulation of Snc1 (Synaptobrevin homolog 1) in the buds of the plasma membrane^[137]^. However, the addition of *C. albicans*-derived EVs restored the resistant effect^[136]^. This implies that the addition of EVs may supplement the required protein that contributes to antifungal drug resistance or vesicle trafficking in fungi. However, further investigation of the EVs’ content is needed. Altogether, these experimental results can indicate the compelling role of fungal-derived EVs in promoting antifungal drug resistance, especially via biofilm formation.

## FUTURE DIRECTION AND CHALLENGES OF EV RESEARCH IN THE FIELD OF UTIs

It is still crucial to explore the role of EVs and the content derived from uropathogens during UTIs. Many research gaps or directions remain to be discovered. Until this stage, almost all studies focused on the protein content inside EVs derived from uropathogens instead of lipids, RNA, and DNA. In the future, researchers can study DNA and RNA content in EVs, and discover if there are any urinary EV biomarkers specific to certain types of uropathogens during UTIs. Moreover, they can investigate the potential molecules or chemicals that can act as EV inhibitors. One method is to focus more on the EVs’ biogenesis in microbes, especially gram-positive bacteria. Recent studies have proposed that the disruption of the peptidoglycan layer caused by the action of prophage promotes EV formation in gram-positive bacteria^[138,139]^. Thus, it can be a direction for the scientists to find which key components control the above pathway and inhibit the EV formation. In addition, testing for any synergistic antimicrobial effects when using the antimicrobial drug in conjunction with the EV inhibitors is also an important research direction. However, the EV inhibitor candidates should mainly target the microbes rather than the human cells. If no such inhibitor is found, a more feasible method is to develop a small interfering RNA drug that targets the pathogenic genes’ expression corresponding to their protein production. Finding a common and consensus target in EVs of bacteria and fungi is better for overcoming multi-species UTIs.

Nevertheless, some challenges exist when scientists and clinicians explore the above research direction. The challenge primarily lies in identifying specific biomarkers, as there is no standardized method to isolate and purify the EVs. At this stage, there are lots of EV isolation and purification methods available, for example, size-exclusion chromatography (qEV columns, a product from Izon Science Limited for performing Size exclusion chromatography), precipitation (ExoQuick and ExoQuick Ultra), iodixanol gradient ultracentrifugation (Optiprep), and affinity-based capture method^[140,141]^. However, different EV isolation and purification methods will co-isolate the impurities, for example, Tamm Horsfall protein (THP) in urine, which entraps EVs and interferes with RNA extraction and miRNA quantification^[142-144]^. Also, studies have shown that THP is a highly glycosylated protein, which can mask the signal of other glycosylated proteins during mass spectrometry proteomic analysis^[145]^. Furthermore, different centrifugation methods and parameters will affect the RNA concentration^[146]^. This may cause bias when examining the abundance of RNA biomarkers in urine during UTIs. Notably, unlike EVs derived from human cells, the distinct biomarker of EVs from many pathogens has not yet been discovered and confirmed, although some studies propose biomarkers for specific pathogens, for example, Sur7 in EVs of *C. albicans* and the Hsp70 domain in nine fungal species^[147,148]^. This further complicates the identification procedure for EV sources. Collectively, more research efforts should be made to tackle the above challenges.

## CONCLUSION

In summary, EVs derived from bacteria and fungi play a significant role in almost every aspect of the pathogenesis of UTIs, including colonization, invasion, immune modulation, immune evasion, and host environmental modulation. They carry different biomolecules that facilitate their persistence, survival, and infection in the urinary system. In addition, given the increasing recognition of the defensive roles of host-derived EVs during infection, scientists should deeply investigate whether there are any potential biomarkers or therapeutic targets for UTIs, rather than focusing solely on pathogen-derived EVs. With a greater understanding of the roles and mechanisms of EVs derived from uropathogens and their hosts, microbiologists and clinicians may identify new therapeutic directions, thereby mitigating the global problem of drug-resistant UTIs.

## DECLARATIONS

### Copyright

© The Author(s) 2025.
